# Development and evaluation of a training module for people with lived experience of mental illness using social contact strategy for stigma reduction: A study protocol

**DOI:** 10.1371/journal.pone.0315618

**Published:** 2025-06-18

**Authors:** Gurucharan Bhaskar Mendon, Santosh Loganathan, Maya Semrau, Anish V. Cherian

**Affiliations:** 1 Department of Psychiatric Social Work, National Institute of Mental Health and Neurosciences, Bengaluru, India; 2 Department of Psychiatry, National Institute of Mental Health and Neurosciences, Bengaluru, India; 3 Centre for Equitable Global Health Research, Department of Global Health and Infection, Brighton and Sussex Medical School, University of Brighton and University of Sussex, Brighton, United Kingdom; 4 Department of Psychiatric Social Work, National Institute of Mental Health and Neurosciences, Bengaluru, India; Western University Faculty of Science, CANADA

## Abstract

Social contact strategy or social contact based anti-stigma intervention, where a person with lived experience (PWLE) of mental illness shares his/her lived experiences with the target group, has been found to be effective in reducing stigma and discrimination. A culturally appropriate social contact based anti-stigma intervention training module would be helpful in training PWLE. Since there is no culturally appropriate training module available in India, there is a need to develop a training module for PWLE of mental illness to deliver a social contact based anti-stigma intervention. Thus, the proposed mixed-methods study aims to develop and test the efficacy of a training module for PWLE of mental illness, using social contact strategy to reduce stigma and discrimination towards people with mental illness amongst undergraduate students. The proposed study will be carried out in three phases; Phase-I: formative work will be conducted using an explorative research design. After a formative and extensive literature review, the culturally appropriate training module will be developed and subsequently reviewed and validated by mental health experts and service users. Phase-II: PWLE of mental illness will be trained using the developed manual adopting a case series design. Phase-III: To test the efficacy of the training, a quasi-experimental research design will be used, in which the target group’s knowledge, attitudes and behaviour towards mental illness will be assessed pre- and post and at three-month follow-up. Socio-demographic data will be analysed using descriptive statistics. Qualitative data (Phase-I and Phase-III) will be analysed through thematic analysis. Based on normality distribution, a parametric test like RMANOVA or an equivalent non parametric test will be adopted during phase III for efficacy testing. In addition, the outcomes amongst the PWLE, i.e., self-stigma and self-esteem, will be assessed and use of the training module will be analysed using thematic analysis.

## Introduction

Stigma is considered to be a sociocultural and interpretive process occurring within a social context [1]. Stigma and discrimination against persons with lived experience (PWLE) of mental illness are universal. However, their manifestations and implications are specific to the context and culture [2,3]. Primarily, self-stigma gives rise to unintended psychological consequences for persons with mental illness, such as decreasing their motivation to pursue goals and increasing/worsening their depressive symptoms [4,5]. Further, social stigma where employers’ fear towards mental illness can lead to the person with mental illness not being hired for a job can result in unemployment, and income inequality [6]. Stigma can also negatively impact help-seeking, treatment adherence, marriage (getting married/marital problems), as person may be fearful about the judgement, social exclusion and negative consequences [7]. Further, it hinders the process of recovery and rehabilitation [8–13]. Anti-stigma interventions can play a significant role in stigma reduction, help in increasing awareness amongst communities, and prevent social exclusion of PWLE of mental illness. Therefore, the development and implementation of anti-stigma interventions are considered to be vital in overcoming stigma and discrimination by the World Health Organisation (WHO) in their Mental Health Action Plan 2013–2020 [14].

Various anti-stigma interventions have come into existence, one of which involves social contact, where a PWLE shares his/her lived experience with the general public or a specific target group. Both direct (in-person, face to face interactions) or indirect (e.g., online, e-contact or imagined contact) have been found to be effective in stigma reduction, due in part to the equal status among the PWLE of mental illness and the general population [15–21]. However, ‘in-person/direct social contact’ is considered the most effective intervention to reduce prejudice, stigma and discrimination, since it allows two-way interaction between the PWLE delivering the intervention and the target group/audience, and the PWLE mental illness taking a lead role that itself is destigmatizing [5,22–26]. Social contact strategies have been found to be effective in the short-term [13,23,24,27,28] and long-term on stigma reduction within several target groups when qualified social contact (i.e., structured, planned, common goal and equal status between PWLE and general public) takes place and the narrations are delivered by PWLE who received training on sharing recovery narration, engagement skills, communication skills, myth-busting, challenging stigma, and handling the disclosure consequences and questions [25,27]. This strategy has also been found to be the strongest proven active ingredient to reduce mental illness related stereotypes and prejudice, and to improve empathy and personal connection with PWLE of mental illness [29,30]. Sharing of lived experiences by people with mental illness can bring about change in the target group both emotionally (e.g., by increasing hope and self-worth) and cognitively (e.g., by improving their knowledge that treatments are available and that there is chance of recovery) [31]. In addition, social contact helps decrease anxiety and increase empathy among individuals by reducing stigma [25,32].

In the context of low- and middle-income countries (LMICs), the evidence on the efficacy of the social contact intervention is thin [24,26,32–34]. Stigma-reduction strategies in LMICs are minimal compared to High Income Countries (HICs) due to inadequate financial resources; for instance, in the form of large-scale anti-stigma campaigns [35,36]. In addition, the components, focus and implementation processes of various anti-stigma interventions have varied based on their feasibility and acceptability in the respective socio-cultural environments in LMICs [37].

According to Gordon Allport, 1954, an equal status between the groups within a situation, common goals, inter-group co-operation and support of authorities and law was considered essential in reducing prejudice during the application of social contact strategy. A well-structured social contact strategy, speaker’s interaction, key message, medium, frequency in intervention, different types of contact, and content for narratives/lived experience shared by the trained person attributes to the quality of social contact [22]. Quality of social contact plays a crucial role in improving relationships between PWLE of mental illness and the general public [1,15,30,33,38–41]. Identifying innovative methods to incorporate social contact within the field of public mental health can significantly help in stigma reduction and discrimination [42].

Selection of the target population within the social contact strategy also makes a difference. ‘Good’ targets are people in positions of power such as employers, landlords, and healthcare providers, faith-based and other community leaders, legislators, schools, entitlement counsellors, and media outlets [43]. Similarly, in India, students, police personnel, traditional healers, primary care workers, nurses, employers, and local community leaders can be considered as relevant target groups for anti-stigma interventions [44]. Contacting target groups involves careful consideration of the venue and timing to increase opportunities for reaching out to larger numbers of the target group.

PWLE are critical in advocating for the elimination of stigma through leading or co-leading positions and interventions in anti-stigma reduction programmes [20]. By involving lived experience in leading and co-leading programmes, an increase in autonomy and resilience can be promoted, and can enhance empowerment and support for PWLE to turn their journey of a mental illness into a positive process of recovery for the public [45].

In India, previous research found that effective stigma-reduction interventions were multi-level, using a combination of intra- and inter-personal and community-level strategies [46]. Even though social contact was the most effective strategy for stigma reduction in India [47,48], there exists minimal information on training PWLE, showcasing a greater need to elucidate and understand the essential components such as skills, content/message and the contextual factors required in developing a training module for PWLE of mental illness who can deliver social contact based anti-stigma interventions in the Indian context. The proposed study aims to fill this gap.

## Materials and methods

**Aim:** To develop and test the efficacy of a training module for people with lived experience of mental illness using the social contact strategy to reduce stigma and discrimination.

### Objectives

- To assess the barriers and facilitators for involving People With Lived Experience (PWLE) of mental illness in the social contact based anti-stigma intervention.
- To develop a module for training people with lived experience (PWLE) of mental illness.
- To test the efficacy of the social contact based anti-stigma intervention (involvement of trained PWLE) with undergraduate students, in terms of knowledge, attitudes, and behaviour.
- To test the efficacy of the training module in people with lived experience (PWLE) of mental illness with respect to self-stigma, self-esteem and use of the training module.

### Methodology

#### Hypothesis.

H_0_: There are no significant differences in knowledge, attitudes and behaviour amongst undergraduate students post assessment compared to pre assessment.

**Fig 1:**
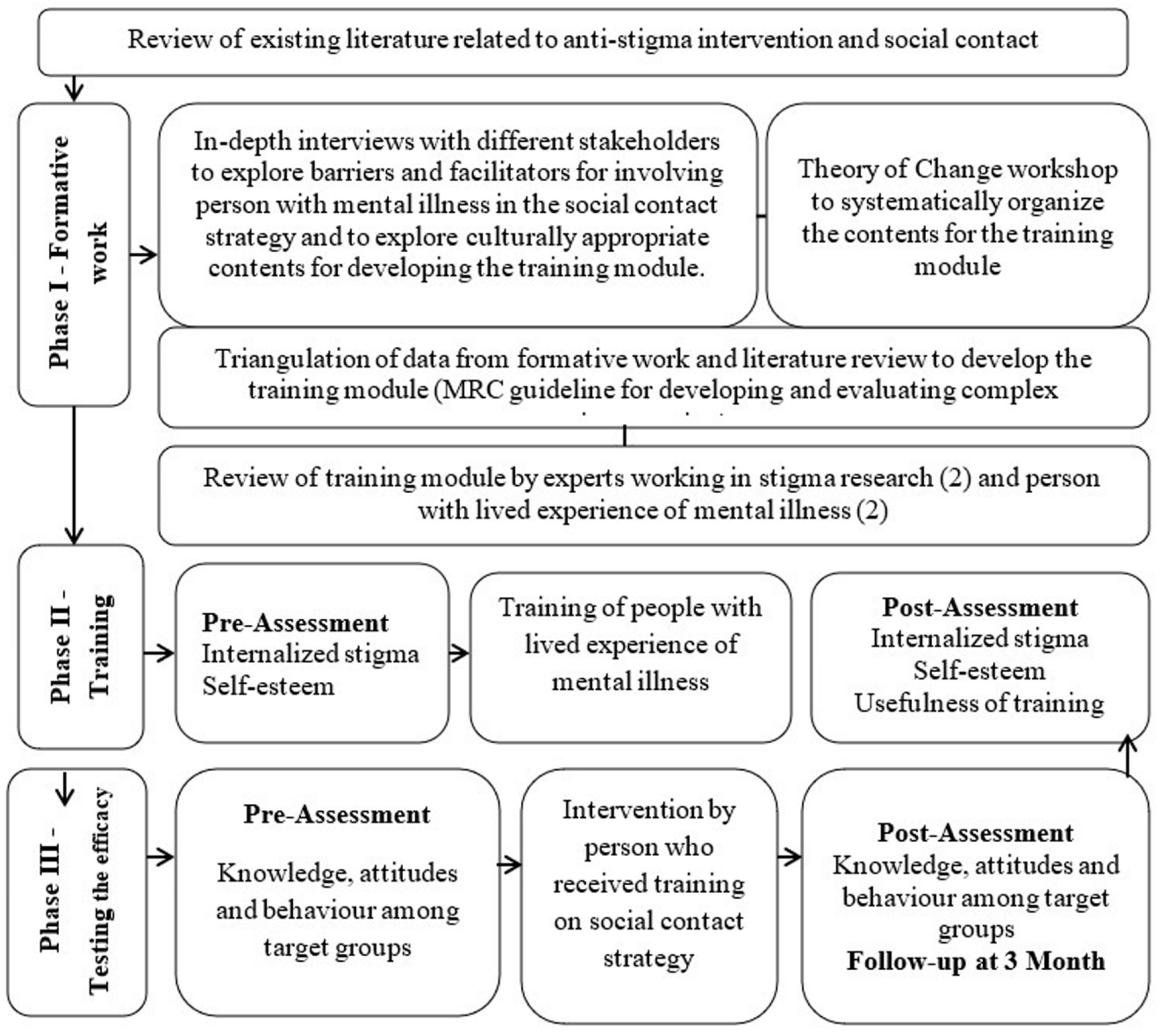
Process of the study.

The proposed study will be carried out in three phases; formative work, training PWLE, testing the efficacy of the intervention (see Fig 1).

#### Research design.

A mixed-methods research design using both quantitative and qualitative methods will be adopted in carrying out the proposed study.

### Phase I – Formative work

#### Research design.

Exploratory research design (qualitative).

Aims;

a) To understand the perspectives of various stakeholders on involving PWLE’s of mental illness in the social contact based anti-stigma intervention.
b) To develop the training module for PWLE’s of mental illness.
c) A Theory of Change workshop will be carried out to systematically organize the content obtained from the stakeholders.

#### Sampling design.

**Sampling:** Purposive sampling will be used to recruit experts and persons with mental illness whereby rich qualitative data from participants will be collected. Hence, a set of inclusion and exclusion criteria has been decided for each stakeholder group to recruit participants and explore their perspective.

**Sample size:** This will be based on saturation of themes, but it is anticipated that the following number of participants would be involved from different stakeholder groups. However, the number of participants may vary.

a) Mental health experts working with persons with mental illness (service providers)from mental health care setting/ private practitioners/persons working in medical colleges/teaching institutes/ District Mental Health Team members (Psychiatrist, Psychologist, Psychiatric Social Worker, Nursing, Psychiatric Rehabilitation member): 10–12 experts across the group based on their expertise in stigma reduction.
b) Non-Government Organizations (NGOs) involved in stigma reduction and application of social contact strategy: 2 experts
c) Service users who are asymptomatic/ recovered from mental illness (common and severe mental illness, and substance use disorder): 5–8
d) Family members/ caregivers of people with lived experience of mental illness: 5–8

**Process:** In phase one, a stakeholder list will be prepared based on the research team’s clinical contacts. The stakeholder list will be prepared based on the inclusion and exclusion criteria, after which the researcher will contact and brief those people about the study through email, telephone call, WhatsApp message or in-person contact. Further, the people who express an interest to participate will be contacted again, detailed information will be shared, and informed consent will be obtained from all stakeholders.

The inclusion and exclusion criteria for the formative work are listed in “S1 Table”, “S2 Table”.

**Tools for data collection:** (Qualitative)

- **Socio–demographic data sheet:** We will develop a socio-demographic data sheet (for service users and service providers) to collect basic details, including personal, family, and social profile of different stakeholders.
- **Semi-structured interview guide:** We will develop three different interview guides to carry out in-depth interviews with service providers, service users and family members. This will help us to explore the barriers and facilitators for involving people recovered from mental illness in the social contact based anti-stigma intervention. Further, this will help us identify and understand the culturally appropriate components for developing the training module.

**Process:** Based on the existing review of literature (the researcher is planning to conduct a scoping review to understand the study area more concretely) and discussion with the experts, we will develop the semi-structured interview schedule. This will be initially reviewed by two peer Ph.D. scholars. Following which, it will be revised and validated by a minimum of two experts working in stigma research. Furthermore, the validated semi-structured interview schedule will be pilot-tested with two members from each stakeholder group (service providers, service users, family member), and any necessary changes/feedback (if any) will be incorporated. After which, the semi-structured interview schedule will be reviewed and finalised.

- **Theory of Change (ToC) framework:** Individuals [8–10] involved in the initial interviews will be engaged in the ToC workshop (No of session: 01). This will help us to identify the culturally appropriate content and systematically organize these contents for the training module. We will adopt the ToC framework from the Medical Research Council UK [49].

#### Statistical analysis:

- Socio demographic data will be analysed using descriptive statistics using R or SPSS software.
- Qualitative data will be analysed through thematic analysis. The six phases framework by Braun & Clarke (2006) will be adopted to identify themes and to develop the training module for person with mental illness: Become familiar with the data, generate initial codes, search for themes, review themes, define themes and write up. The thematic analysis will be done by using NVIVO/atlas.ti software.

The data collected from the formative work and literature reviews will be triangulated to develop the training module for people with lived experience of mental illness. The developed training module will be shared with mental health experts [2] and people with lived experience of mental illness for review and content validation. The reviewers’ suggestions will be incorporated and the module will be revised accordingly. This training module will be used for training the people with lived experience of mental illness.

**Phase II – Training of people with lived experience (PWLE) of mental illness and pilot-test:** A case series design will be adopted to assess self-stigma, self-esteem and usefulness of the training module.

#### Sampling design.

**Population:** People with lived experience of mental illness, currently asymptomatic/recovered, motivated and willing to participate.

**Sampling:** Purposive sampling will be used to recruit people with lived experience of mental illness (common and severe mental illness, and substance use disorder).

**Sample size:** 2–4 participants will be recruited and trained (the sample size will be decided considering the practical experience of similar work carried out by supervisors).

The inclusion and exclusion criteria for phase-II are listed in “S3 Table”.

#### Process of participants’ recruitment.

After the initial process of approaching individuals in regular outpatient services at tertiary care, interested and motivated individuals will be identified and considered for the study. The participants’ potentiality will be discussed within the research team. Participants will all be given the opportunity to carry on with their regular follow-up with the respective treating team.

#### Tools for data collection.

a) **Qualitative interviews** will be carried out with participants to gather their views and perspectives on the application of the developed module. Researcher will develop and validate an interview guide to collect qualitative data through in-depth interviews.
b) **Quantitative assessment:** Scales details mentioned in “S4 Table” will be administered with PWLEs who attend the training at pre- post training (after carrying out the intervention with the target group).

#### Statistical analysis.

- Socio-demographic data will be analysed using descriptive statistics.
- Qualitative data will be analysed through thematic analysis using NVIVO/atlas.ti software.
- For the quantitative data, mean scores of scales will be analysed using R software to test the efficacy of the training module amongst people with lived experience of mental illness with respect to self-stigma and self-esteem.

**Tentative Training Module Outline:** The draft training module will be based on the existing evidence. Components of the training module for people with lived experience of mental illness in social contact strategy consist of illness experience, treatment experience, recovery stories and positive views towards mental illness. Existing literature [1] consists of key domains like; 1) speaker: person who has lived experience in mental health and currently recovered and those who are ready to share their lived experience with the target group/audience. 2) Message which consists of details on treatment experience, correcting misperception/ misunderstanding about mental illness, sharing recovery stories and resource details. 3) Interaction strategies including communication, positive response and myth busting.

**Tentative number of sessions:** Approximately 5 sessions. (Each session- 60–90 minutes depending on the outcome of the formative work)

**Session venue:** To be decided based on the place of study and feasibility.

**Trainer:** The researcher will be carrying out the training sessions.

**Training language:** Training will be provided in the local language of Karnataka, i.e., Kannada.

### Process of training

**Pre-test:** The researcher will assess the study participants’ self-stigma and self-esteem.

Training (Individual/ group) will be carried out with participants considering the situation.

Training PWLE of mental illness.

**Post-test:** The researcher will assess the participants’ self-stigma and self-esteem using the mean values of the scales mentioned in “S4 Table”. Acceptability, appropriateness and feasibility of the training module will be measured using qualitative interview schedules. Post-test of the trained person will be carried out after the intervention programme delivered to the target group.

**Follow up:** Follow-up assessment of the trained persons will be carried out using the above-mentioned scales (“S4 Table”) after 3 months.

**Pilot-test:** The persons who received the training will be further involved in carrying out the anti-stigma intervention with the undergraduate students to pilot test the feasibility of carrying out the intervention and the appropriateness of the test measurement scales. The pilot test will be carried out with 20 undergraduate students from the one college of Ramanagara based on convenient sampling (10% of participants representing the target group). Their knowledge, attitudes and behaviour will be assessed using the scales mentioned in “S6 Table”. Feasibility test of the intervention will be carried out using the Acceptability of Intervention Measure (AIM), Intervention Appropriateness Measure (IAM), and Feasibility of Intervention Measure (FIM). Following which, suggestions and feedback will be incorporated to the training module and intervention content. This will be reviewed before testing the efficacy.

**Phase III –Testing the efficacy:** A mixed-methods quasi-experimental research design with pre – post-test will be adopted.

### Sampling design

#### Population.

The selection of the target population in the social contact  makes a difference. ‘Good’ target groups are people in positions of power such as employers, landlords, healthcare providers, faith-based and other community leaders, legislators, schools, entitlement counsellors, and media outlets [43]. In India, students, police personnel, traditional healers, primary care workers, nurses, employers and local community leaders can be considered as target groups in anti-stigma interventions [44]. Based on discussions with experts working in the area of stigma reduction and suggestions from the institutional review board, we chose undergraduate students as target group, to test feasibility. Further, we believe that positive attitudinal change about mental illness among this group is helpful for society.

#### Undergraduate students.

**Sampling:** Multi-stage sampling will be used to select undergraduate students in Phase III.

**1**^**st**^ **level:** Selection of 1–2 colleges from Ramanagara taluk using simple random sampling.

**2**^**nd**^ **level**: Selection of course from selected college using simple random sampling.

**3**^**rd**^ **level:** Selection of class from selected course using simple random sampling.

**4**^**th**^ **level:** Selection of students from selected class using cluster sampling.

The inclusion and exclusion criteria for undergraduate students during phase-III are explained in “S5 Table”.

#### Tools for data collection.

a) **Qualitative assessment (**Post Assessment): We will prepare an interview guide (validated by two experts working in stigma reduction), which will be used to assess the target group participants’ views and feedback (e.g., whether the trained person is able to manage the session, able to communicate, share their lived experience etc) on the session led by the person trained in social contact strategy.
b) **Quantitative assessment:** Scales details mentioned in “S6 Table” will be administered with two target group participants at pre-post and three month follow up.

#### Statistical analysis.

- Socio-demographic data will be analysed using descriptive statistics.
- Qualitative data will be analysed through thematic analysis to identify participants’ views and feedback on the intervention session led by the trained person.
- Qualitative analysis will be carried out using NVIVO/ atlas. ti software.
- Quantitative data will be analysed using R software. Based on normality distribution, a parametric test like Repeated Measures of ANOVA (RMANOVA) or an equivalent non-parametric test will be carried out to test the efficacy of the developed module.

**Process of Phase-III,**

**Pre-test** Knowledge, attitudes and behaviour will be assessed using the appropriate scales.

**Intervention** Approximately 3−4 sessions (Each session- 1hour) within the period of two months. (The number of social contact sessions can be shortened depending on the outcome of the formative phase as mentioned)

**Post-test** Knowledge, attitudes and behaviour will be assessed using the scales mentioned in “S6 Table” one month after the session. Qualitative measures such as acceptability of session, attitudes, participants’ feedback on session, and skills of the person who carried out the session will be assessed.

**Follow-up:** Knowledge, attitudes and behaviour will be assessed at 3 months follow up.

**Target population:**

**Table:** 

Study Phase	Target group	Involvement
Phase-I (Formative work)	Service providers: Mental health professionals (psychiatrist, psychologist, psychiatric social worker, psychiatric nurse) from varied settings	Participate in interviews, which explore their perspective on involvement of PWLE in social contact and develop content for training module. Further, they will participate in Theory of Change- (ToC) workshop.
	Service users diagnosed with common or severe mental illness or substance use disorder	
	Family member or primary caregivers of service users	
Phase-II (Training PWLE)	Service users (PWLE)	PWLEs receive the training on disclosing lived experience and being involved in social contact.
Phase-III Testing the efficacy (Awareness and social contact with audience)	Service users (PWLE)	Trained PWLE will be the resource person for the social contact
	Audience group	Undergraduate students will be audience who will have social contact with PWLE and listen to the PWLE’s lived experience narration.

### Ethical considerations

- The study purpose and procedure will be explained to each participant and a participant information sheet will be provided. Following which, the written informed consent will be obtained.
- The researcher will ensure confidentiality by anonymising the individual’s identity during the academic presentation/publication. We will ensure that all data will be coded with the record ID against participants name, no personal data will be revealed to anyone outside the study team. All audio records, transcripts and data will be stored with the record IDs rather than participants’ name.
- The data collection and interviews will be carried out at a venue that is comfortable to participants.
- A common rule will be set and followed by the researcher and the participants that at any point of time they will not judge the other participants with respect to the experiences shared by them.
- Participants’ rights in the due course of the study will be given prime importance. They will be given complete right to withdraw from the study at any point of time.
- Participant’s difficulties (emotional) and queries/doubts will be considered and the researcher will provide adequate support and necessary information regarding the same.
- Consent will be sought before the audio recording. The recordings will be kept in a password-protected drive which can be accessed only by the researcher. Recordings will be deleted after the analysis and thesis submission.
- As the trained persons will be delivering the intervention to the target group there will be chance for the confidentiality issues, wherein they will be sharing their lived experiences. But to avoid any further issues, the trained person will be asked to provide consent for sharing their experiences with the target group before the session.

## Discussion

Involving PWLE of mental illness in social contact will be a research partnership which helps to reduce stigma towards mental illness. As per existing literature, there are possibilities of negative consequences of PWLEs’ involvement which will be mitigated by appropriate training of PWLEs and involvement of mental health professionals. Content of the training module or delivery of the training will be determined only after the formative work. The anti-stigma intervention approach during phase-III may vary; for instance, the intervention could be led by a service user alone or it could be led by a researcher and service user in partnership depending on the findings from the formative work. The study will be implemented in a rural or semi urban place, so replicating it in an urban set up may be easy or difficult. Sustainability of involvement of PWLE’s stands as a question considering the socio-cultural aspects of the country.

### Implications of the study

- A culturally and contextually relevant training module will be more effective.
- Training module for social contact would reduce the self-stigma and increase the self-esteem among person who received the training.
- Trained person can be utilized as the resource person for the anti-stigma intervention.
- A registry for individuals trained in delivering social contact can be maintained. This can help the NGOs working for stigma reduction connect with the trained individuals and collaborate in working for stigma reduction.

## Supporting information

S1 Table Phase-I, Formative work inclusion criteria for different stakeholders. (DOCX)

S2 Table Phase-I, Formative work exclusion criteria for different stakeholders. (DOCX)

S3 Table Phase-II, Inclusion and exclusion criteria for PWLE. (DOCX)

S4 Table Scales used for assessment with PWLE at Phase-II. (DOCX)

S5 Table Inclusion and exclusion criteria for undergraduate students in phase-III. (DOCX)

S6 Table Scales used for assessment with undergraduate students at Phase-III. (DOCX)
