# Supplementary material for: Development and evaluation of a training module for people with lived experience of mental illness using social contact strategy for stigma reduction: A study protocol
Source: PLoS One. 2025 Jun 18;20(6):e0315618. doi: 10.1371/journal.pone.0315618 (PMC12176174; doi:10.1371/journal.pone.0315618)
Supplement: S1 Table — (DOCX) [file pone.0315618.s001.docx]

| **Inclusion criteria** | | |
| --- | --- | --- |
| **Service providers** | **Service users** | **Family members** |
| Participants aged between 30 and 60 years. | Participants aged between 20 and 60 years. | Primary caregiver of PWLE who has been with the person for a minimum of one year. |
| Participants working in the area of mental health for at least 10 years. | Participants who can communicate either in Kannada or English |  |
| Participants who can communicate either in Kannada or English | Participants who are asymptomatic /recovered from (common and severe) mental illness/with lived experience. |  |
| Participants from various backgrounds will be chosen: medical colleges/ teaching institutes/ hospitals/ private practitioners/ Non- Government Organizations (NGOs) | Ability to engage in discussion/ open-minded and functional individuals |  |
| Participants who are interested and motivated. | Participants who are interested and motivated. |  |

**Table- 1** (Phase-I, Formative work inclusion criteria for different stakeholders**)**
