# Supplementary material for: Development and evaluation of a training module for people with lived experience of mental illness using social contact strategy for stigma reduction: A study protocol
Source: PLoS One. 2025 Jun 18;20(6):e0315618. doi: 10.1371/journal.pone.0315618 (PMC12176174; doi:10.1371/journal.pone.0315618)
Supplement: S2 Table — (DOCX) [file pone.0315618.s002.docx]

**Table- 2** (Phase-I, Formative work exclusion criteria for different stakeholders**)**

| **Exclusion criteria** | | |
| --- | --- | --- |
| **Service providers** | **Service Users** | **Family member**s |
| Person below 30 years and above 60 years of age. | Person who is symptomatic/ cognitively impaired/unable to engage in interview. | Primary caregiver who has not spent a minimum of one year with the person with mental illness. |
| Person working as service provider in mental health care setting with less than 10 years of work experience. | Person with intellectual and developmental delay. | Family members with intellectual developmental delay/ mental illness / cognitive impairment. |
