# Supplementary material for: Development and evaluation of a training module for people with lived experience of mental illness using social contact strategy for stigma reduction: A study protocol
Source: PLoS One. 2025 Jun 18;20(6):e0315618. doi: 10.1371/journal.pone.0315618 (PMC12176174; doi:10.1371/journal.pone.0315618)
Supplement: S3 Table — (DOCX) [file pone.0315618.s003.docx]

**Table- 3** (Phase-II, Inclusion and exclusion criteria for PWLE**)**

| **Inclusion criteria** | **Exclusion criteria** |
| --- | --- |
| Participants will include persons recovered from mental illness. (Clinical Global Impressions (CGI) Scale score)) | Persons who are symptomatic/ cognitively impaired. |
| Participants who can communicate in Kannada and English. | Person with intellectual developmental delay. |
| Participants who are interested and motivated. | Person scoring more than 3 in CGI (CGI-Severity & Improvement). |
