# Supplementary material for: Development and evaluation of a training module for people with lived experience of mental illness using social contact strategy for stigma reduction: A study protocol
Source: PLoS One. 2025 Jun 18;20(6):e0315618. doi: 10.1371/journal.pone.0315618 (PMC12176174; doi:10.1371/journal.pone.0315618)
Supplement: S4 Table — (DOCX) [file pone.0315618.s004.docx]

**Table- 4** (Scales used for assessment with PWLE at Phase-II**)**

| **Scale name** | **Used in Indian context** | **Assessment** |
| --- | --- | --- |
| Internalized Stigma of Mental Illness (ISMI)-Ritsher et al., 2003 | James, T. T., & Kutty, V. R. (2015) | Pre - post test and follow up. |
| Rosenberg Self-Esteem Questionnaire (Rosenberg, 1965) | Pal et al.,2017 | Pre - post test and follow up. |
