# Supplementary material for: Development and evaluation of a training module for people with lived experience of mental illness using social contact strategy for stigma reduction: A study protocol
Source: PLoS One. 2025 Jun 18;20(6):e0315618. doi: 10.1371/journal.pone.0315618 (PMC12176174; doi:10.1371/journal.pone.0315618)
Supplement: S5 Table — (DOCX) [file pone.0315618.s005.docx]

**Table- 6** (Inclusion and exclusion criteria for undergraduate students in phase-III**)**

| **Inclusion criteria** | **Exclusion criteria** |
| --- | --- |
| Participants within the age range of 18 - 21 | Individuals not falling in the age range of 18-21 will be excluded. |
| Participants who can understand Kannada or English. | Individuals with language barriers. |
| Participants on regular degree course. | Individuals on distance learning/course |
