# Supplementary material for: Development and evaluation of a training module for people with lived experience of mental illness using social contact strategy for stigma reduction: A study protocol
Source: PLoS One. 2025 Jun 18;20(6):e0315618. doi: 10.1371/journal.pone.0315618 (PMC12176174; doi:10.1371/journal.pone.0315618)
Supplement: S6 Table — (DOCX) [file pone.0315618.s006.docx]

**Table- 6** (Scales used for assessment with undergraduate students at Phase-III**)**

| **Scale name** | **Used in Indian context** | **Test** |
| --- | --- | --- |
| **Police constable trainees/ Undergraduate students** | | |
| Mental Illness Knowledge Schedule (**MAKS**) (*1-6 Questions only)* | De Silva et al., 2016, quoted in Pingani et al., 2019 | Pre & Post, Follow up-3 month |
| Reported and Intended Behaviour Scale (RIBS) | INDIGO Network Team | Pre & Post, Follow up-3 month |
| Community attitude towards mental illness (CAMI) | Ahuja et al., 2017 | Pre & Post, Follow up-3 month |
